# On the traces of XPD: cell cycle matters - untangling the genotype-phenotype relationship of XPD mutations

**DOI:** 10.1186/1747-1028-5-24

**Published:** 2010-09-15

**Authors:** Elisabetta Cameroni, Karin Stettler, Beat Suter

**Affiliations:** 1 Institute of Cell Biology, University of Bern, Baltzerstrasse 4, CH-3012 Bern, Switzerland

## Abstract

Mutations in the human gene coding for XPD lead to segmental progeria - the premature appearance of some of the phenotypes normally associated with aging - which may or may not be accompanied by increased cancer incidence. XPD is required for at least three different critical cellular functions: in addition to participating in the process of nucleotide excision repair (NER), which removes bulky DNA lesions, XPD also regulates transcription as part of the general transcription factor IIH (TFIIH) and controls cell cycle progression through its interaction with CAK, a pivotal activator of cyclin dependent kinases (CDKs). The study of inherited *XPD *disorders offers the opportunity to gain insights into the coordination of important cellular events and may shed light on the mechanisms that regulate the delicate equilibrium between cell proliferation and functional senescence, which is notably altered during physiological aging and in cancer.

The phenotypic manifestations in the different *XPD *disorders are the sum of disturbances in the vital processes carried out by TFIIH and CAK. In addition, further TFIIH- and CAK-independent cellular activities of XPD may also play a role. This, added to the complex feedback networks that are in place to guarantee the coordination between cell cycle, DNA repair and transcription, complicates the interpretation of clinical observations. While results obtained from patient cell isolates as well as from murine models have been elementary in revealing such complexity, the *Drosophila *embryo has proven useful to analyze the role of XPD as a cell cycle regulator independently from its other cellular functions. Together with data from the biochemical and structural analysis of XPD and of the TFIIH complex these results combine into a new picture of the XPD activities that provides ground for a better understanding of the patophysiology of *XPD *diseases and for future development of diagnostic and therapeutic tools.

## Review

Evolution often strives for simplification; it may be advantageous for organisms to preserve resources by using few molecules to fulfill many different tasks, in particular when these functions are spatially or temporally separated. But on the flip side of the same coin, reducing redundancy implies the hazard of multiple consequences upon malfunctioning of a single key element. The multitude of disease phenotypes associated with defects in the multitask protein XPD perfectly illustrates this concept. XPD is a DNA helicase involved in at least three crucial cellular mechanisms: repair of DNA damage by nucleotide excision repair (NER), transcription and cell cycle regulation.

### XPD disorders

**Figure 1:**
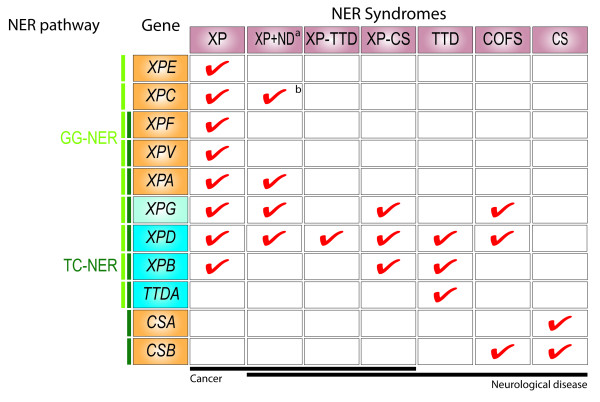
**The genetic causes of the different NER syndromes are depicted on this table**. XP, xeroderma pigmentosum; XP+ND, xeroderma pigmentosum with neurological disease; TTD, trichothiodystrophy; XP-TTD, complex syndrome with phenotypic manifestations of both XP and TTD; COFS, cerebro oculo facial skeletal syndrome; CS, Cockayne syndrome; XP-CS, complex syndrome with phenotypic manifestations of both XP and CS. ^a ^Includes the cases described as DeSanctis Cacchione (DSC) syndrome. ^b ^Only in rare cases. Mutations in genes encoding components of the GG-NER pathway (light green) cause the XP syndrome or the one of the three complex syndromes XP+ND, XP-TTD and XP-CS, all of which are characterized by increased cancer incidence. Mutations in genes specifically involved in the TC-NER branch (dark green) are associated with CS or the more severe COFS syndrome. Mutations affecting the XPD, XPB or TTDA subunits of the TFIIH complex (blue), which intervenes in a common step of NER, affect both pathways in addition to transcription and lead to TTD. Reflecting the central role of TFIIH in the common steps of NER, in transcription and in regulation of the cell cycle, mutations in its components (and in particular of XPD) are found in most of the syndromes.

Mutations in the human gene *ERCC2/XPD *can give rise to six distinct genetic disorders with a broad variety of phenotypes collectively called NER syndromes [1-3]. Aside from *XPD *mutations, mutations in genes encoding other components of the NER pathway can also cause these phenotypes. Therefore we will refer here to *XPD *disorders to describe the phenotypical manifestations of mutations in the gene coding for XPD (Figure 1). One of the most striking phenotypes shared by these syndromes is the premature appearance of some, but not all, aging-related features (termed segmental progeria) such as marked cancer predisposition, photoaging of skin and eyes and progressive tissue deterioration due to either replicative senescence of stem cells or apoptosis of terminally differentiated cells [4-6].

**Figure 2:**
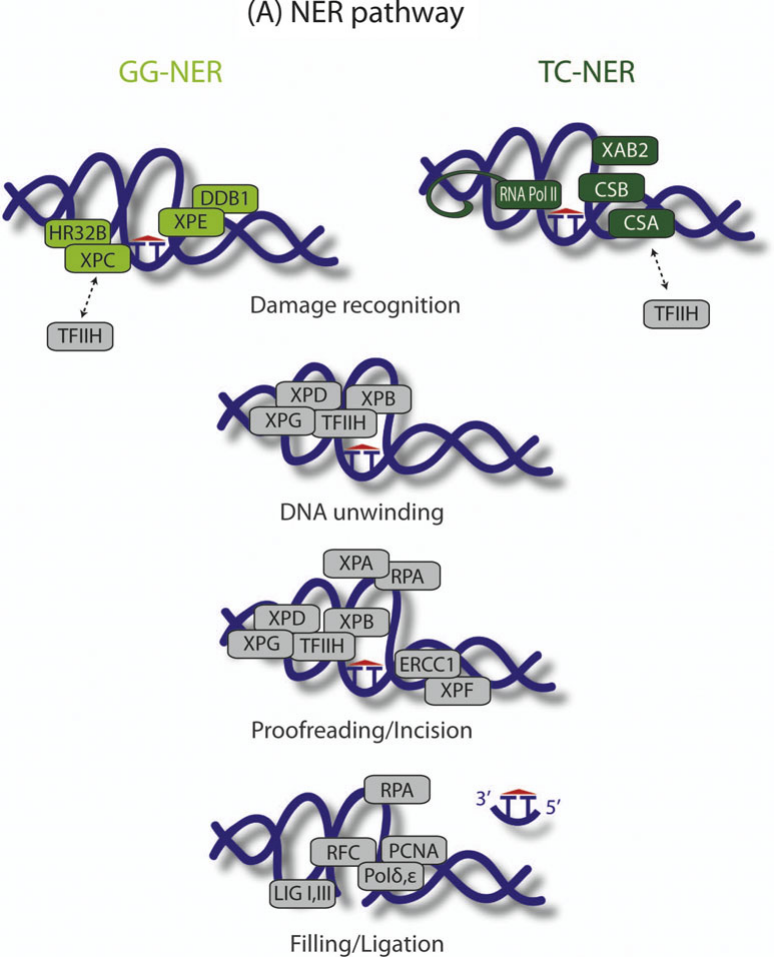
**Nucleotide excision repair (NER) repairs UV-induced pyrimidine dimers, intra-strand and protein-DNA crosslinks, and a wide range of bulky chemical adducts **[11]. Its two subpathways global genomic repair (GG-NER) and transcription-coupled repair (TC-NER) differ in their damage recognition. DNA distortions anywhere in the genome are recognized by XPC. Lesions that cause only minor distortions, such as UV-induced cyclobutane pyrimidine dimers (CPD) are recognized by the damage DNA binding complex (DDB) composed of DDB1 and DDB2 (encoded by the XPE gene). DDB induces a strong kink in the DNA, enhancing its recognition by XPC. The XPC and DDB complexes are dispensable for the TC-NER subpathway that relies on RNA Pol II for the recognition of DNA damage. When RNA Pol II encounters a lesion that blocks its progression, it recruits CSA, CSB and XAB2. Either XPC in GG-NER or CSB and CSA in TC-NER then recruit further NER factors, including the transcription factor TFIIH to the damage site. The XPD and XPB subunits of TFIIH (depicted separately to point out their essential role in NER) unwind the DNA around the lesion. This process requires the helicase activity of XPD and the ATPase (but not the helicase) activity of XPB. XPG stabilizes the interaction between TFIIH and XPD. The opened denaturation bubble is stabilized by the XPA complex (XPA and the heterotrimer RNA), which also participates in the proofreading step (verification of the presence of a lesion and identification of the damaged strand). The damaged strand is incised on the 3' side of the lesion by XPG and on the 5' by the ERCC1-XPF heterodimer. The gap left by the excised oligonucleotide is filled by one of the replicative DNA polymerases (Pol δ or Pol ε) using the undamaged strand as a template and sealed by either ligase I or III.

Patients suffering from *xeroderma pigmentosum *(XP) are sensitive to sunlight, even a limited UV exposure leads to the premature onset of skin abnormalities resembling those associated with normal aging. Skin abnormalities in XP patients range from pigmentation defects (dyspigmentation, freckling) to the development of multiple benign (seborrheic keratoses, actinic keratoses) and neoplastic (squamous cell carcinoma, basal cell carcinoma and melanoma) cutaneous lesions [7]. Occasionally, XP patients develop also progressive ocular disease and eye cancer. Moreover they are generally more prone to some internal tumors, particularly of the lungs and the gastro-intestinal tract. The median age of onset of malignant neoplastic disease is around 8 years of age for skin neoplasm, and with this well below that expected for the general population (50 years for US), while the frequency of its occurrence is one- to several-thousand times greater than in the general population [8]. Most of the mutations causing XP affect genes encoding components of the NER pathway (Figure 2). Additional XP mutations are found in *XPV*, which encodes a DNA polymerase involved in translesion DNA synthesis [9]. Primary skin fibroblasts isolated from XP patients show a drastic reduction in the capacity to perform DNA-repair and are hypersensitive to the killing effects of UV-irradiation [10]. Around 20% of patients show a severe XP phenotype with neurological abnormalities emerging at the age of 10-20 years. Neurological symptoms are mainly due to progressive neurodegeneration and add to the characteristic UV-sensitive XP phenotype [11]. Some, but not all, of the cases presenting neurological defects meet the definition of the *De Sanctis-Cacchione syndrome *(DSC), the maximal form of neurological involvement [8]. These cases have been grouped as a distinct syndrome named XP with neurological disease (XP+ND) [2].

Mutations in the *CSA *and *CSB *genes specifically impair the mechanism of transcription-coupled repair (TC-NER) - one of two branches of the NER pathway (Figure 2) - giving rise to *Cockayne Syndrome *(CS) [12]. Similar to XP patients, CS patients show an elevated sensitivity to sun light, but pigmentation abnormalities and skin malignancies are absent. Patients have a reduced stature, malformed bones and retina, and mental retardation mainly due to degeneration of the white matter [1,13]. Common segmental progeroid features in CS are: the loss of subcutaneous fat tissue (cachexia), sensorineural deafness, retinal degeneration, white matter hypomyelination and CNS calcification [3]. Mutation in three of the XP genes, *XPB*, *XPD *and *XPG *can give rise to combined phenotypes of XP and CS (XP-CS) with neurological dysfunctions reminiscent of CS combined with the typical skin abnormalities of XP, including increased cancer incidence [14,15]. Strikingly, primary fibroblasts from XP-CS patients are considerably more sensitive to killing by UV irradiation than cells from XP patients.

Mutations in *XPD *may also cause *trichothiodystrophy *(TTD), this definition encompasses several diseases, which share the hallmark of sulfur-deficient brittle hair exhibiting a typical tiger tail pattern under polarized light [16,17]. Many TTD patients also display enhanced sun-sensitivity, but as in CS the typical pigmentation defects and the cancer predisposition observed in XP are absent [1]. Interestingly, even though skin fibroblasts from TTD patients show enhanced UV-sensitivity, NER activity in these cells ranges from acutely defective to nearly normal with only about half of the cases displaying marked NER defects. This apparent absence of correlation between NER activity and severity of the disease suggests that the phenotypes are not exclusively determined by the impact of the *XPD *mutation on NER [18,19]. Additional TTD traits include progressive cognitive impairment, abnormal face shape and reduced body size [1,14,20]. Progeroid features in TTD patients are similar to those observed in CS and XP-CS patients, including demyelinating neuropathy and brain calcifications. In addition to the *XPD *and *XPB *genes, *TTDA *- encoding a structural component of the TFIIH complex - has also been associated with TTD. Patients displaying combined features of XP - such as dry skin, skin cancer, mental and physical impairment - and of TTD - such as ichthyosis and the typical sulfur-deficient and tiger-tail patterned hair - are assigned to the combined XP-TTD syndrome group [21]. This complex clinical manifestation has so far been associated exclusively with mutations in *XPD*.

Finally, the allelic *XPD *combination R681N and R616W gives rise to the *Cerebro-oculo-facial-skeletal syndrome *(COFS). Patients show microcephaly, dysmyelination, defects in neuronal migration and innervation, affecting both the grey and the white matter. Additionally, patients show severe mental retardation, drastically reduced lifespan and often prenatal growth failure [3,14,22]. The features of the disease are very similar to CS although patients show more severe eye and brain defects and fibroblast lines isolated from these patients are more sensitive to the killing effect of UV irradiation than cells isolated from CS patients [22]. The COFS syndrome was first described with mutated *XPG *and *CSB *genes and was proposed to be a severe allelic form of CS [23].

### *XPD *phenotypes: insights from structural data

Due to the implication of XPD in fundamental processes such as transcription, NER and cell cycle regulation, malfunctioning of this enzyme results in pleiotropic phenotypes. Understanding how different mutations in the same gene, often changing adjacent aminoacids, differentially affect the functionality of XPD requires profound knowledge of the molecular structure of XPD and of the complexes in which it is integrated.

#### Structure of the XPD-containing complexes

XPD bridges the core subcomplex of the general transcription factor IIH (core TFIIH) to the CDK activating kinase (CAK) complex, which also exists as a free trimeric complex with a distinct function [24,25]. An additional quaternary CAK-XPD complex with unknown biological relevance has been isolated from mammalian cells [26] as well as from *Drosophila *embryonic extracts [27]. Recently, a novel XPD complex has been described that is distinct from the TFIIH complex; in this MMXD (MMS19-MIP18-XPD) complex, XPD is found associated with MMS19, MIP18 (formerly FMA96B), Ciao1 and ANT2 but no additional TFIIH component [28].

The TFIIH complex is currently the best characterized XPD complex. It is made up of 10 subunits and defects in three of them are known to cause NER syndromes; namely the 5'-3' helicase XPD, the 3'-5' helicase XPB and TTDA/p8, a 8kDa protein required for stabilization of the complex [29]. In addition to its helicase activity, TFIIH possesses also kinase activity, which is provided by the cyclin-dependent kinase 7 (CDK7) and is required for the transactivation of various transcription factors as well as for the regulation of RNA Polymerase II (RNA Pol II) [30-35]. Importantly, the substrate specificity of CDK7 is dependent on the molecular context; when CDK7 is integrated in the TFIIH complex, it phosphorylates the C-terminal domain of RNA Pol II in addition to various transcription factors, while as part of the free CAK complex it shows higher activity towards CDKs such as CDK1, CDK2, CDK4 and CDK6 [25,36].

Although additional protein-protein interactions with XPB, p62 and p44 have been proposed to mediate CAK binding to core TFIIH [25], reduced binding of the XPD component most drastically affects the composition and stability of the complex. Analysis of CAK localization and activity in *Drosophila *suggested a model in which XPD, besides anchoring CAK to TFIIH, also acts as a molecular dispatcher allocating the activity of the CAK/CDK7 kinase to the appropriate cellular substrates [37,38]. Additionally, despite not being a TFIIH subunit *per se*, the NER protein XPG was also found to associate with the TFIIH complex by interacting directly with XPD stabilizing the interaction between TFIIH and the CAK-XPD complex [39,40]. Remarkably, as observed for *XPD*, mutations in *XPG *also lead to XP or XP-CS phenotypes, suggesting that part of these phenotypes are related to a reduced capacity to anchor CAK to TFIIH resulting in increased free CAK activity.

**Figure 3:**
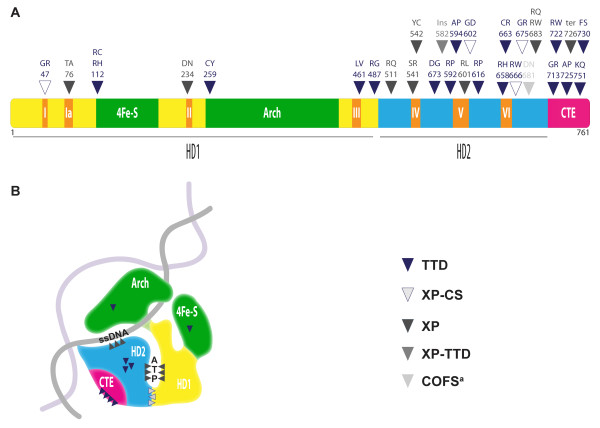
**(Adapted from **[42]) **(A) Linear aminoacid sequence of XPD, the positions of some of the (putative) causative mutations found in XPD syndromes are indicated **[1,10,13,42,45,117]. Alterations considered to be completely inactivating [10] are not shown on this figure. With two exceptions (an aminoacid insertion at position 582 leading to XP-TTD and a non-sense mutation at position 726 leading to XP) all the mutations mapped in this drawing cause aminoacid substitutions. The phenotypic manifestation of these mutations is indicated by the color of the arrow as described in the legend. ^a ^COFS is caused by the allelic combination R616W (also found in TTD patients) and D681N (B) Cartoon representing the 3D structure of XPD according to the solved crystal structures of two bacterial homologues [42,45], the C terminus (pink) is absent in the bacterial proteins and therefore its location on the cartoon is not based on crystallography data [42]. Arrowheads represent schematically the predominant localization of mutations in XPD giving rise to the different disorders. XP mutations are found mainly in the vicinity of the helicase motifs on residues that form the channels binding either ATP or ssDNA. XP-CS mutations are at the interface between the two helicase domains and affect functional flexibility of the enzyme. In contrast TTD mutations appear to increase the flexibility of the XPD molecule affecting its framework stability and thereby also important intermolecular interactions such as those required to form the TFIIH complex. Additional TTD mutations are located in the C terminal part of the molecule outside of the catalytic core, Most of these mutations abolish interaction with the TFIIH subunit p44 [35].

The function of XPD appears to be modulated by interaction with the TFIIH complex as full helicase activity requires the interaction with the TFIIH subunit p44 and possibly also other subunits [35]. The functional relevance of the XPD-p44 interaction is underlined by the high proportion of disease-causing mutations found in the C-terminal region of XPD which is required for this interaction [41](Figure 3A). Mutations in *XPD *preventing interaction with p44 as well as mutations in *XPD *or other TFIIH subunits (*e.g*. TTDA/p8) that affect the stability of the core TFIIH complex have been suggested to lead to TTD [42]. Interestingly, in a *Drosophila *TTD model carrying TFIIH-destabilizing mutations in the genes coding for the XPB or the p52 subunit, overexpression of TTDA/p8 suppresses accumulated developmental defects associated with these mutations [43].

The structure of human TFIIH complex has been resolved by electron microscopy, revealing that the core TFIIH subunits fold into a ring structure with a bulge formed by the CAK component. XPD locates at the base of this bulge and appears to be integrated into the ring structure opposite to XPB relative to the bulge and both XPD and XPB are bound to p44 [44]. While CAK is required for the function of TFIIH and may have a role in stabilizing the complex, it is clearly dispensable for formation of the core TFIIH as this can be formed *in vitro *in the absence of the CAK components [44]. From this observation it was proposed that XPD may be added on top of a preexisting ring, a hypothesis that is compatible with the hypothesis of a dynamically regulated interaction between the CAK-XPD and the core complex [37].

#### 3D-structure of XPD

While the helicase activity of XPD is essential during NER, the role of XPD in transcriptional regulation depends, rather than on its enzymatic activity, on its ability to bridge the CAK to the TFIIH core complex. Similarly, a scaffolding role has also been proposed for XPD in the regulation of the cell cycle, where XPD is thought to dynamically regulate the association of CAK with TFIIH [37]. The crystal structures of the XPD catalytic core from *Sulfolobus acidocaldarius *and *Thermoplasma acidophilum *have recently been resolved [42,45]. Mapping the different disease-related point mutations in *ERCC2/XPD *on these 3D structures provided better understanding about how such mutations could impact the catalytic activity of XPD, its framework structure, as well as its ability to undergo correct protein-protein interactions with TFIIH and CAK components (Figure 3).

XPD is a member of the superfamily 2 (SF2) DNA helicases, but unlike most SF2 members it unwinds DNA duplexes with 5'-3' polarity. The catalytic core of XPD is comprised of two helicase domains, HD1 and HD2, which contain 4 and 3, respectively, of the 7 conserved helicase motifs (Figure 3A). Two additional accessory domains are inserted into HD1: a 4Fe-S cluster and an Arch domain [46]. The 4FeS cluster domain, conserved among related SF2 helicases, is inserted in the N-terminal helicase domain (HD1) between motifs Ia and II that are equivalent to the Walker A and B motifs of many ATPases. Besides contributing to the overall stability of the enzyme, this cluster provides residues involved in the intramolecular interactions that form the DNA binding interface and thus is thought to be critical for helicase activity [42]. In addition, it forms a pocket that is ideally positioned to accommodate the damaged DNA strand during the step of damage proofreading in NER [45]. Intimate connection to the ATP binding site (Figure 3B) suggests that the conformation of the 4Fe-S cluster may be regulated by ATP hydrolysis. Moreover, the redox-sensitive binding of the four cysteine residues of the cluster with four Fe ions may provide an additional mode of regulation of the enzyme's conformational states, which are likely to be critical for interaction with other partners. The Arch domain forms an arch-shaped structure that joins the HD2 motor helicase domain to the ATP-helicase domain and forms a small interface with the 4FeS cluster to make an enclosed tunnel, positioning of the unwound DNA within this tunnel is likely to be relevant for the presentation of the damaged site to the NER machinery components [42].

With the exception of 6 mutations - five of which cause TTD - positioned in the C-terminal extension of the HD2 domain, most of the disease causing point mutations in *ERCC2/XPD *map within its catalytic core (Figure 3A). According to their predicted impacts on DNA or ATP binding, XP mutations tend to alter residues that form the DNA- and ATP-binding channels, XP-CS mutations are located at the HD1-HD2 interface and decrease the functional flexibility of the enzyme, which is essential during DNA repair. Mutations causing TTD are generally impacting the framework stability of the enzyme, which in turn is likely to disturb the interactions with the other components of the TFIIH complex.

### Insights from the molecular functions of XPD and its complexes

#### Nucleotide excision repair

Several extrinsic factors like radiation, sunlight and chemicals but also various intrinsic factors such as free radicals of metabolic origin, mechanical stress and DNA replication can alter important biomolecules including, and perhaps most importantly, nucleic acids. Failure to repair damage to DNA can lead to cell death or cancer. Thus, several distinct repair pathways are in place to preserve the genetic information: homologous recombination, mismatch repair, DNA strand crosslink repair, nonhomologous end-joining, base excision repair (BER) and nucleotide excision repair (NER) [47]. The latter requires the enzymatic activity of XPD and will thus be the focus of the following paragraph. NER is elicited by chemically- and UV-induced helix-distorting lesions, including cyclobutane pyrimidine dimers (CPD) and 6-4 photoproducts (6-4PP) [48]. In addition, NER can also recognize and repair small oxidative adducts.

NER is a sophisticated DNA repair mechanism entailing damage recognition and proofreading, incision on both sides of the lesion followed by removal of a 20-30 nucleotide long DNA fragment, DNA re-synthesis and ligation. Approximately 30 proteins are involved in one or both of two NER subpathways: namely the global genome repair (GG-NER ) and the faster transcription-coupled repair (TC-NER), which differ in the initial, damage-recognition steps (Figure 2). In GG-NER, repair of helix-distorting base damage in the entire genome, is initiated with damage recognition by the XPC-HR23B complex. For mildly distorting lesions the access of XPC is facilitated by XPE. The TFIIH complex is subsequently recruited through interaction with XPC. In contrast, TC-NER is confined to actively transcribed DNA regions and is elicited by stalling of the transcribing RNA Pol II at the site of damage. The stalled polymerase is recognized by the *CSB *gene product, this allows the subsequent access of CSA. Then TFIIH is recruited by CSA and CSB along with XPG to remodel the RNA Pol II complex. After damage recognition, both pathways recruit sequentially the other components of the repair machinery. The TFIIH helicases XPB and XPD mediate DNA unwinding then XPA-RPA contributes to the stabilization of the opened helix by binding to the single-stranded DNA together with XPG. In addition XPA and the TFIIH subunits XPB and XPD are involved in recognition of the damaged strand and proofreading of damage [49]. Correct positioning of the two endonucleases XPG and XPF-ERCC1, which catalyze the incision of the DNA 3' and 5' of the damage, requires XPA-RPA. The DNA polymerases δ and ε fill up the resulting gap using the undamaged strand as template and ultimately a ligase (either I or III) is recruited to seal the nick [7,50].

While the role of the core TFIIH complex and of XPD in NER is well established, it is not clear to what extent the CAK subcomplex is required for this function; even though the CAK subcomplex is recruited to the damaged site, the kinase activity of CDK7 appears to be dispensable for repair [40]. Moreover, following repair initiation XPA catalyzes the release of CAK from the TFIIH complex to favor the binding of additional NER proteins thereby accelerating NER [51]. Strikingly CAK has also been shown to negatively regulate XPD unwinding activity *in vitro*, suggesting that its release is a prerequisite for efficient repair [7,50,52].

#### Transcription and RNA Pol II pausing

XPD and the CAK subcomplex are both required for full transcriptional activation *in vitro*, yet genetic and biochemical data indicate that neither the helicase activity of XPD nor the kinase activity of CDK7 are essential for this function. Mutations corrupting the helicase activity of XPD did not impair the ability of TFIIH to promote transcription *in vitro *[41,53]; similarly, a TFIIH complex containing a kinase-inactive CAK was shown to support *in vitro *transcription [53-55]. Moreover a temperature sensitive allele of *CDK7 *was compatible with adult survival in *Drosophila*, further indicating that CAK activity may be dispensable for basal transcription [56]. These observations led to the assumption of a merely structural role of CAK during transcription. However, it is important to point out that most if not all the studies addressing the effects of XPD and CDK7 mutations on transcription relied on *in vitro *transcription assays limited to a restricted number of promoters. Thus, it cannot be excluded that the enzymatic activities of XPD and CAK are required for transcription activation *in vivo*. In particular, while basal transcription may indeed not require CAK, this complex is still likely to be involved in the regulation of transcription of a subset of inducible genes.

TFIIH contributes to transcription at several early stages of the transcription cycle. While the most obvious contribution is opening of the transcription bubble via helicase activity of XPB, TFIIH also directly interacts with a variety of transcription activators and repressors that deliver signals to, and receive signals from, the transcription machinery. In addition, CDK7-dependent phosphorylation of the carboxy-terminal domain (CTD) of the largest subunit of RNA Pol II is required, along with the action of the pause factors DSIF and NELF, for proper promoter proximal pausing and 3' pausing [30]. Polymerase pausing enables the recruitment of factors involved in mRNA capping as well as of additional proteins that contribute to the transcription and processing of RNAs in a gene-specific manner. At promoters with paused polymerase productive transcription elongation can be rapidly triggered by CDK9/PTEFb, which phosphorylates DSIF and NELF as well as the CTD of RNA Pol II. Importantly, some transcription factors activate transcription at the level of elongation by mediating the recruitment of CDK9 to the paused initiation complex. In mouse embryonic stem cells the transcription factor c-MYC was demonstrated to exploit this strategy to activate gene transcription [57]. Direct modulation of RNA Pol II pausing both at the 5' and 3' end of genes is emerging as an important regulatory mechanism in particular for genes involved in development or genes that need to be rapidly co-regulated in response to stimuli. Intriguingly, this is in line with the observation that CDK7 activity is essential during embryogenesis and gametogenesis as well as for mounting transcriptional responses to environmental stress such as heat shock [30,57-61].

Transcriptional changes affecting particular genes or groups of co-regulated genes, rather than reduced levels of basal transcription, may underlie some of the NER-independent phenotypes caused by *XPD *mutations. The transcriptional profiles of cell lines isolated from TTD and XPD patients revealed alterations of only a relatively small subset of the transcriptome, including down- and up-regulated genes [62,63]. In addition, the XP- and TTD-type mutations analyzed in these studies affected transcription of the downregulated genes to a similar extent, indicating that the transcription defect is probably not a prerogative of TTD as previously believed. Remarkably, a mutant cell line derived from a cancer-prone XP-CS patient presented a high number of overexpressed genes, mainly encoding proteins that are involved in the regulation of cell proliferation, differentiation and transformation [62]. Thus transcriptional alterations may also contribute to the increased susceptibility to carcinogenesis in XP and in the combined syndromes.

Although part of the transcriptional changes accompanying *XPD *mutations are certainly caused by the cellular stress generated by defective NER, the finding that CAK is involved in the regulation of RNA Pol II pausing raises the possibility that some mutations in *XPD *may affect preferentially the transcription of genes that strongly rely on this mechanism for timely and coordinated expression. Another intriguing, poorly explored possibility is that mutant *XPD *alleles display differences in their ability to undergo protein-protein interaction with specific transcription factors.

#### Cell cycle regulation by the CAK complex

Cyclin dependent kinases regulate the cell cycle, integrating activating signals and signals from various checkpoints that ensure that each event of the cell cycle occurs when the previous phase has been successfully completed. The activity of CDKs is controlled in a tightly timed manner by the availability and levels of cyclins, by the association of specific regulators and by various phosphorylation and autophosphorylation events. Aside from its involvement in gene transcription and NER, XPD is also a regulator of cell proliferation. XPD affects cell cycle progression directly by regulating CDK7, a critical activator of CDKs. Amongst the most relevant targets of CDK7 for the cell cycle are the cyclin-dependent kinases CDK1, CDK2, CDK4 and CDK6. Phosphorylation by CDK7/CAK is necessary for full activation of these kinases, which subsequently promote the phosphorylation of key cell cycle substrates.

Reducing CDK7 protein levels in *Drosophila *by the use of the conditional *cdk7*^*ts1 *^allele disclosed mitosis as the cellular process with the highest requirement for CDK7 activity, being the first to be affected at the restrictive temperature [56,64]. Interestingly, reduction by half of the gene dose of *XPD *resulted in a partial rescue of the *cdk7*^*ts1 *^phenotype, indicating that mitotic progression is facilitated by the lowered XPD levels [37]. Because XPD anchors CAK to TFIIH, the simplest explanation is that reduced XPD levels release more CAK in a form that has more access to and/or more activity towards CDK7's cell cycle targets. At least in surviving animals such reduced XPD levels do not appear to be detrimental for transcription activity, which is again consistent with transcription requiring less CDK7 kinase activity than mitosis and with the recessive nature of the *XPD *mutant phenotypes that implies that one functional copy of *XPD *is sufficient. Moreover, in embryonic insect cell lines RNAi knock down of *XPD *resulted in increased rates of cell division without obvious reduction in cell volume, indicating that XPD is not limiting for bulk transcription as the residual levels of XPD were sufficient to sustain growth of hyperproliferating cells. Overexpression of XPD in fly embryos also indicated a cell cycle inhibitory function of XPD as it resulted in cell cycle arrest at different phases and even caused skipping of mitosis. The observed phenotypes indicate that elevated XPD levels lead to an inactivation of mitotic CDK activity possibly because XPD acts a molecular dispatcher for CDK7 by sequestering it in the form of a TFIIH-CAK complex, or possibly a XPD-CAK complex, and thereby hampering the phosphorylation of CDKs. Indeed, staining of embryos expressing XPD from the strong heat shock promoter revealed aberrant XPD and CDK7 accumulation at ectopic sites in the cytoplasm [37]. Remarkably, regulatory mechanisms seem to be in place to maintain constant cellular levels of XPD, as overexpression of a XPD construct did not result in the expected high levels of the protein product (our unpublished data). Thus, ectopic localization of XPD and perhaps CDK7/CAK may reflect sites of proteolytic degradation. Consistent with regulated XPD degradation, XPD was shown to interact with several components of the proteasome in a yeast four hybrid assay [52].

**Figure 4:**
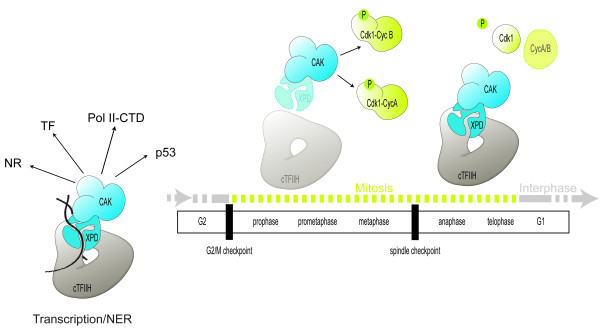
**Model for the role of XPD as a molecular dispatcher directing CAK localization according to the cellular requirements for transcription, DNA repair and mitotic progression**. During transcription and repair XPD is tightly associated with the TFIIH complex and recruits the CAK complex, which is necessary for the phosphorylation of different substrates such as nuclear receptors (NR), transcription factors (TF), the C-terminal domain of RNA Pol II (Pol II-CTD) as well as proteins involved in the DNA damage responses including p53. During the cell cycle XPD levels seem to fluctuate in some cell types. At prometaphase reduced levels of XPD (or altered affinities) allow the release of CAK from the core TFIIH complex (cTFIIH). Free CAK promotes mitotic entry by facilitating phosphorylation of the T-loop of the mitotic kinase CDK1, which is necessary for the formation of functional Cyclin-CDK complexes. Mitotic exit can occur only upon downregulation of mitotic cyclins, which may require prior dephosphorylation of the CDK1 T-loop. During exit from mitosis and interphase or during DNA-damage induced cell cycle arrest, high levels of XPD may prevent persistent activation of the mitotic kinase by relocating CAK away from its mitotic substrates.

In *Drosophila *blastoderm embryos and cultured cells, XPD is redistributed and possibly down-regulated in the early part of mitosis [37]. Thus, under physiological conditions the oscillating levels of cellular XPD may determine the localization and the fate of the CDK7-CAK complex and thereby the phosphorylation of the different CDK7 substrates (Figure 4). As the bridging subunit of the TFIIH-CAK complex XPD is pivotal in orchestrating the processes of mitosis and transcription, as well as mitosis and repair, in a mutually exclusive manner. During interphase (or in response to DNA damage) higher levels of XPD ensure the integrity of the TFIIH complex supporting transcription (or repair). At the same time XPD prevents improper cell division by sequestering CAK. In contrast, during mitosis the integrity of the TFIIH complex is not critical whereas phosphorylation of CDK1, which is performed by the CAK complex without core TFIIH, is essential [38]. Reduced levels of XPD during mitosis may then allow the release of sufficiently high amounts of free CAK for proper phosphorylation of CDKs.

Mutations in *XPD *that modify the interaction of the CAK (or the XPD-CAK) subcomplex with the core TFIIH may result in dissociation of free CAK complex that may then acquire higher affinity for cell cycle substrates such as CDKs. In addition, reduced association of the CAK subunit also disturbs the functionality of the TFIIH complex itself. According to the model described in Figure 4, such mutations are likely to lead on one hand to increased mutagenic rates and misregulated transcription and on the other hand to enhanced proliferation. The latter is achieved at the expense of checkpoints and accuracy. This, together with the elevated chances of unrepaired DNA damage, increases the chance of cells becoming tumorigenic. In contrast, mutations that affect the conformation of XPD and thereby the stability of TFIIH, like the ones found in the cancer-free TTD syndrome, are thought to impact the function of TFIIH in transcription and repair. Intuitively, reduced stability of the TFIIH complex would be expected to result in increased levels of free CAK, and thus increased cancer risk, analogously to reduced XPD levels. However, various reports indicated that destabilization of the TFIIH complex caused reduced levels of all measured components, including the CAK subunits, thus leaving no excessive free CAK that would promote over-proliferation [65].

A quaternary complex containing all three CAK subunits plus XPD was described in *Drosophila *embryos as well as in mammalian cells [27]. Its biological relevance, however, remained unknown. One possibility is that XPD sequesters CAK into an inactive quaternary complex, independent of the remaining TFIIH core. If this is true, mutations that affect only the interaction of XPD with TFIIH, but retain efficient binding to CAK, are expected to be found in patients showing characteristics of the cancer free XPD disorders. If in contrast this XPD-CAK complex represents an active form of the CAK complex, then such *XPD *mutations are expected to result in sustained phosphorylation of mitotic substrates and thus to be associated with increased cancer incidence in the human disease.

#### XPD regulation of mitotic kinase

The phenotypes resulting from the modulation of XPD protein levels clearly indicate that XPD controls the cell cycle by functioning as a molecular dispatcher that directs the subcellular localization of CAK and thereby defines its substrate availability. However, it is not trivial to isolate the cell cycle functions of XPD from its other functions. An experimental setting where transcription and NER are not needed, would allow to specifically address the cell cycle role of XPD. Such a situation is found in the young *Drosophila *embryo. *Drosophila *embryos lacking XPD show several mutant phenotypes, including general loss of division synchrony, problems in spindle targeting, and numerous mitotic defects. Mitotic defects appear to be mediated by a loss of CAK control and ensuing deregulation of mitotic kinase activity as they can be partially rescued by reducing the levels of the limiting CDK1 partner Cyclin B (CYCB) [66]. In the absence of XPD, embryos show increased tendency to improperly segregate chromatin. They display aberrant nuclear figures with chromatin bridges and loss of chromosomal material, indicative of a strong chromosome instability phenotype.

The activity of the mitotic kinase CDK1 is required to enter mitosis and its downregulation at the anaphase transition is a prerequisite for mitotic exit. Embryos lacking XPD build up CDK1 activity normally as individual nuclei enter mitosis. In contrast, its downregulation and with it mitotic exit are delayed. While in normal embryos CDK7 is progressively removed from the chromosomes during mitotic exit, in the absence of XPD excessive localization of CDK7 is seen on anaphase chromosomes, this is reflected by the persistence of phosphorylated histone 3 over the entire chromosomes through ana- and sometimes even telophase, indicative of mitotic kinase hyperactivity [66]. It is currently not clear how prolonged chromosomal CDK7/CAK activity would prevent CDK1 inactivation, as this process depends mainly on CYCB degradation. One possible explanation emerging from the results by Li and colleagues [66] is that lack of XPD and misregulated CAK prevent this degradation possibly due to excessive T-loop phosphorylation of CDK1, which allows and stabilizes the interaction with CYCB [64,67]. A simple explanation would be that M phase cyclins are more easily degraded if they are only loosely associated with CDK1. This model would then also suggest that CDK1 normally gets dephosphorylated in its T-loop to allow efficient and rapid cyclin degradation, a pre-requisite for normal exit from mitosis. This view is challenging the initial assumption that T-loop phosphorylation is not dynamic, however it is supported by evidence for a more dynamic process both in *Drosophila *embryos and in human cells [37,56,64,68]. Conversely, for mitotic entry to occur CDK1 phosphorylation by CAK is required to promote the formation of a stable CDK1-CYCB complex [69]. Cyclin B binding and phosphorylation by CAK are essential CDK1 activation steps and both steps occur in a concerted manner in the cell. The phenotype of the embryos lacking XPD now suggests that at the end of their round of duty the two processes are linked again and that T-loop dephosphorylation may even be required for CDK1 inactivation and possibly Cyclin B destruction [66]. Indeed, there is evidence that dephosphorylation of the CDK1 T-loop is part of the normal mitotic exit in young *Drosophila *embryos during the later nuclear division cycles because phosphorylation is reduced during interphase [70]. Furthermore, a requirement for dephosphorylation of the T-loop Thr of CDK1 was also described for fission yeast mitotic exit, where overexpression of a phosphomimetic CDK1^T161E ^causes cell division defects in anaphase [71]. The mode of regulation summarized in Figure 4 is proposed based on the analysis of a peculiar embryonic cell cycle, which consists only of alternating S and M phases with no requirement for transcription. It remains to be tested whether this model holds true also in a complete diploid cell cycle. Interestingly, reduction of XPD in human cells also causes spindle and chromosome segregation defects [28]. However a possible connection to the regulation of mitotic kinases has not been investigated.

#### Role in checkpoint activation

DNA damage and replication checkpoints, induced during interphase, maintain genome stability by delaying cell cycle progression to allow time for DNA damage repair to occur or for DNA replication to be completed. DNA damage may also induce apoptosis, actively killing cells when repair did not occur or the damage was too severe. This safety mechanism eliminates potentially deleterious mutants from a cell population. Which checkpoint cascade is activated depends on the cell cycle stage, on the type of damage and on the intervening repair pathway [72-74].

The checkpoint cascade triggered by the type of DNA damage repaired by NER culminates with the inhibition of CDKs. Briefly, DNA lesions are recognized by a network of sensor and mediator factors that result in the rapid recruitment of the PI3K-related kinase ATM-Rad3 related (ATR) to the site of damage, this kinase then activates CHK1, which ultimately leads to cell cycle arrest by impinging at various level on the Cyclin-CDK system (reviewed in [72-74]). The response to DNA damage consists of a rapidly induced transient cell-cycle delay (the CDC25 branch), which is eventually replaced by a slowly operating sustained cell cycle arrest (the p53 branch). Phosphorylation by CHK1 leads to ubiquitination and proteasome-mediated degradation of the CDK-activating phosphatase CDC25. Inactivation of CDC25 prevents the removal of inhibitory phosphorylation on CDKs and causes a transient cell cycle delay. In addition DNA damage repair kinases target p53 ultimately leading to the transcriptional activation of various genes that are part of a p53 transcriptional program. The p53 response requires several hours as it depends on the accumulation of p53 and of its targets, in particular the CDK inhibitor p21. In contrast to the transient CDC25 response the p53 response can be protracted and, if the lesions are too extensive, p53 can predispose the cells to apoptosis by upregulating the expression of the proapoptotic proteins BAX and MDM2. Thus the p53 branch constitutes an important antitumoral barrier that prevents the expansion of cells bearing unrepaired DNA damage. If on the other hand the damage is removed promptly and the repair events completed, p53 becomes ubiquitinated and the cell-cycle arrest is relieved. Thus the outcome of the repair process - be it successful repair or the failure to remove the damage - has to be communicated to the checkpoint pathway in order to promote cell cycle resumption or cell death.

Initial processing of the DNA lesions by NER is necessary to allow checkpoint complexes to bind stably to UV-damaged chromosomes in order to monitor the repair process and sustain the cellular responses. Defects in components of the GG-NER pathway and in various TFIIH subunits (*i.e*. *TTDA*, *XPB *and *XPD*) result in totally or partially defective checkpoint activation upon UV irradiation [29]. In contrast, the TC-NER subpathway is dispensable for checkpoint activation and indeed patients carrying mutations in TC-NER-specific genes present a strong epidermal cytotoxic response upon UV-exposure which probably prevents precancerous skin lesions from developing into cancerous lesions [75].

The primary function of the DNA-damage checkpoint is to allow for efficient DNA repair, both by delaying the cell cycle and by sustaining the activity of the DNA repair mechanisms. In ATR-dependent phosphorylation of XPA promotes enhanced XPA activity and thereby increases the efficiency of NER and cell survival upon UV irradiation [76]. Also the early events of the p53 response promote repair in part by promoting the expression of GG-NER-specific factors, such as the XPE binding partner DDB1 [77]. In addition, after the different components of NER have assembled correctly at the site of damage, inhibition of the helicase activity of XPB and XPD by p53 is thought to block the translocation of the TFIIH along the DNA, thus favoring the formation of a stable complex and with this the precise removal of the damage [78].

Failure to repair UV-induced DNA damage triggers p53-mediated apoptosis, interestingly also this response was shown to require functional XPD and XPB helicases, further suggesting that mounting of the NER response is a prerequisite for full activation of the p53 pathway [79]. Furthermore, TFIIH may also contribute to the sustained p53 response to persistent DNA damage via its kinase activity, as p53 is a substrate for phosphorylation by the CAK complex [80].

#### Regulation of MYC expression and activity

TFIIH exerts growth control also by regulating the expression levels of the transcription factor MYC, as this was reported to be deregulated in *XPD- *and *XPB*-deficient human cell lines [81,82]. Notably, MYC has a conserved role in coordinating many essential processes, including proliferation, growth, differentiation, metabolism and apoptosis. As a consequence of its pivotal role as a growth regulator, even slight disturbances of MYC expression directly influence the size and growth rate of cells in culture and *in vivo*, where oncogenic activation of MYC occurs in a variety of tumors [83]. This raises the intriguing hypothesis that deregulation of MYC expression may be connected to the increased cancer proneness of XP patients. MYC exerts its function by controlling the expression of nearly 10% of the whole genome; it binds to a E-box motif in the 5' region of target genes and promotes transcription elongation by recruiting P-TEFb/CDK9, which in turn releases the poised RNA Pol II complex [57]. Binding to the consensus sequence requires heterodimerization of MYC with either MAX or MAD proteins, and the interaction partner determines whether MYC will act as a transcriptional activator or repressor. In addition, MYC was shown to generally influence chromatin structure by promoting both ATP-dependent remodeling of nucleosomes and histone acetylation, the two major chromatin modifications involved in the regulation of transcription [83].

As a powerful positive regulator of growth, MYC imposed the evolution of molecular constraints to ensure that its synthesis and activation only occur at the proper moment. At steady state the levels of the MYC transcript as well as of the encoded protein are short-lived and scarce. The promoter of MYC is subject to tight and rapid regulation at the level of transcriptional elongation by the antagonistic action of the transcriptional activator FUSE-binding protein (FBP) and the FBP-interacting repressor (FIR). Interestingly, the function of FIR and FBP requires the helicase activity of TFIIH [84]. *XPB *and *XPD *mutations appear to simultaneously affect activation and repression of MYC transcription, interfering with the fine-tuning of cellular MYC levels. The effect of these mutations are highly variable and while some cells or tissues may experience reduced MYC activity and thus reduced proliferation, elsewhere the overexpression of this oncogene may trigger cancer [81].

Also the posttranslational regulation of MYC may involve TFIIH. MYC stability and function are controlled by a chain of interdependent phosphorylation events, initiated by phosphorylation on a serine residue by a proline-directed protein kinase such as MAPK or CDK1. This phosphorylation stabilizes MYC and promotes its activity, but in addition it provides the docking site for the GSK3 kinase. Phosphorylation of MYC by GSK3 initiates a series of regulatory events eventually leading to the degradation of MYC [85]. The interaction of TFIIH with CAK, controlled by XPD, determines the substrate specificity of CDK7 and thus the activation state of CDK1, which in turn regulates the early events of the described MYC cycle. Thus XPD is involved in the regulation of MYC levels both at the transcriptional and the posttranscriptional levels. Deregulated MYC activity by itself may not lead to cancer as MYC, besides proliferation, also increases p53-mediated apoptosis by suppressing anti-apoptotic proteins when the level of growth factors are low. Hence a second hit mutation impairing the apoptotic pathway, or a single mutation affecting both pathways, is necessary for MYC-driven tumorigenesis to occur [86]. Furthermore XPD and XPB activities are required for both MYC regulation and p53-mediated apoptosis [79,82]. These mutations are therefore candidate for such a tumorigenic mutation as they simultaneously increase cell proliferation and reduce apoptosis.

In addition to transcriptional regulation, MYC can also impact on mRNA translation by promoting cap methylation. MYC-dependent cap methylation occurs independently of transcription and does not require binding to E-box elements in the promoter region [87]. Interestingly translation of *CDK7- *and *CDK9-*encoding mRNAs is boosted by MYC-induced cap methylation [88]. It appears therefore that the activities of MYC and XPD converge on CDK7, antagonistically regulating its activity, expression and spatial localization. Experiments in mice fibroblasts demonstrated that MYC can also affect cell cycle progression by positively regulating CYCD and its cognate CDKs, CDK4 and CDK6, and by inhibiting expression of their inhibitors p27 [89] and p15^INK4b ^[90]. Notably, CDK4 is also a target of CAK, thus CDK7 and MYC cooperatively impinge on cell cycle progression by regulating common targets as well as each other.

### A complicated genotype-phenotype relationship

A direct cause-effect relationship between inefficient DNA repair and predisposition to tumors is not sufficient to explain the elevated occurrence of cancer manifested in XP-D patients since the cancer free COFS and TTD syndromes are accompanied by similar defects in repair. Neither can NER deficiency alone explain all the clinical phenotypes in TTD such as the characteristic brittle hair and the various neurological symptoms, which are likely to involve also damage-independent developmental processes, sensitive to transcription alterations or to improper cell cycle regulation. Several hypotheses have been formulated to explain the complicated genotype-phenotype relationship of mutations in the *XPD *gene. Notably, the hypotheses discussed here are not mutually exclusive and it is likely that different events concur in the definition of the complex *XPD *phenotypes.

#### The transcription defect hypothesis

The transcription defect hypothesis postulates that the TTD and CS phenotypes are caused by subtle alterations of transcription, due to reduced cellular levels of stable TFIIH complexes [91]. This model also implies that the absence of cancer in TTD, COFS and CS patients is due to the reduced levels of basal transcription that probably does not support the accrued protein synthesis requirement of the fast growing tumor cells or, alternatively, to the reduced transcription of one or several factors required for tumor progression. Reduced basal transcriptional levels were observed using *in vitro *transcription assays and *in vivo *transcription of a reporter gene. Results from these studies showed that *XPD *mutations found in TTD patients, but not those found in XP patients, strongly impaired the ability of a purified TFIIH complex to sustain transcription of the reporter [41]. However, this view is challenged by DNA microarray studies showing that TTD and XP mutations affect basal transcription to similar levels in human fibroblast cell lines [62,63]. Interestingly, although they were limited to few disease-related point mutations, these studies revealed distinct transcriptional signatures of UV-irradiated human TTD, XP and XP-CS cell lines with both up- and down-regulated genes. The cancer predisposition in XP may also be explained with upregulation of cell-cycle genes.

#### The type-of-damage hypothesis

Due to differences in metabolism and because not all tissues are equally exposed to exogenous damaging agents (for example the skin is more exposed to UV than internal tissues), different cell-types display different lesion spectra and thus put different demands on DNA repair and damage responses. In addition, different repair mechanisms are induced in different phases of the cell cycle [72], implying that postmitotic and mitotic tissue are different in terms of the kind of damage they accumulate and of the cellular response elicited by this damage. Depending on the specific NER subpathway they affect (TC-NER or GG-NER), different mutations in NER genes may more strongly affect specific tissues and thus cause specific diseases. Defective DNA repair also leads to progressive degeneration of cells not exposed to UV. This has been attributed to inadequate repair of oxidative damage that results from normal metabolism. It is currently not known if and how much of this physiological damage is repaired by NER and would thus be affected by mutations in *XPD*. For example neuronal death can be triggered by relatively low levels of damage (probably of oxidative nature) in transcribed genes that result in RNA Pol II stalling. As this kind of damage is subject to TC-NER it is less efficiently removed in patients with mutations in genes encoding TC-NER components. Consistent with this model, XPC and XPE patients, who retain proficient TC-NER, generally do not display neurological symptoms [11]. On the other hand, there is good evidence in support of a deficiency in repair of oxidative damage being the cause of the progressive deterioration in CS, which may be particularly significant for tissues with high oxygen demand such as the brain [92]. Can this explanation be applied to explain the different syndromes associated with distinct mutations in *XPD*? As depicted in Figure 2, XPD is involved in a common step of TC-NER and GG-NER, after damage recognition. Thus, it may be expected to affect both pathways in equal measure. However, mutations in *XPD *do not always lead to neurological symptoms. One possible explanation compatible with this model would be that mutations in *XPD *lead to neurological defects if they affect the ability of the TFIIH complex to be recruited by TC-NER-specific damage-recognition complexes.

In addition to preventing damage repair, mutations in genes encoding components of the NER pathway may affect DNA damage signaling. Failure to activate the checkpoint cascades contributes to genomic instability, resulting in increased propensity to cancer. In contrast, mutations that affect only the repair function of NER genes are less likely to cause cancer, as the cell death responses are still in place. Instead they may lead to increased tissue deterioration as a secondary effect of a proficient damage-induced senescence mechanisms [93].

Persistent signaling of the DNA damage checkpoint leads to cell death. This can be circumvented by the activation of the mechanisms of translesion DNA synthesis by either error-free or error-prone DNA polymerases. Replication through photoproducts requires the sequential action of Polη (encoded by *XPV*) and Polζ which are recruited by PCNA when this is monoubiquitinated in response to stalling of the replication fork [94]. Boyle *et al*. directly assayed the repair of UV-induced damage in cells isolated from different patients and found that TTD cells in comparison to XP cells remove DNA damage at a normal rate, despite having low TFIIH levels and impaired XPD helicase activity. Interestingly, they observed a delayed recruitment of repair proteins to the site of damage in both XP and TTD cells; but while in TTD cells the reduced TFIIH complex stability facilitates the dissociation of the repair proteins from the damaged DNA, repair proteins persisted at the site of damage in XP cells, presumably hampering the access of the error-free DNA polymerase η, encoded by *XPV*, and thus increasing genomic instability [95].

In cells from XP-CS patients the NER machinery readily detects UV-induced DNA damage and can initiate repair. However, aberrant incisions are observed at undamaged sites. It has been proposed that TFIIH exists in either a transcription competent or a repair competent conformation. Upon receiving a signal from the damaged DNA, TFIIH may become activated into its repair mode and recruited to the site of damage. If the functional switch does not occur, there is no repair. If the switch occurs in the absence of damage, this leads to aberrant NER activation. Indeed, in XP-CS cells the mode switch occurs promptly, but TFIIH is not relocated from the sites of transcription, resulting in incision in the actively transcribed DNA rather than at the lesion site. On one hand this increases the mutation rate upon UV-exposure (causing the XP phenotype) and on the other hand it may reduce gene expression due to the introduction of DNA breaks at the sites of transcriptional initiation (causing the CS phenotypes) [96].

#### The cell-cycle hypothesis

The function of XPD as a regulator of proliferation may be responsible, at least partially, for the increased incidence of neoplastic diseases in XP patients [37,38]. The proposed model (Figure 4) postulates that high levels of XPD negatively regulate cell-cycle progression by retaining the CAK complex in the transcription and repair competent TFIIH complex. Under normal conditions, at mitosis or after DNA repair, cell-cycle progression would be favored by reduction of XPD levels or its affinity to CAK, resulting in increased levels of a free form of the CAK complex and thus in higher affinity for CDK substrates.

According to this model, whether or not *XPD *mutations result in increased proliferation and cancer depends on the affinity of the mutated protein for the other subunits of the TFIIH complex as well as on the stability of the *XPD *protein product. Mutations resulting in a less stable protein or in a protein with reduced ability to sequester the CAK complex, preventing it from performing its cell cycle function, are expected to lead to increased CAK activity towards its CDK substrates and thus to increase the proliferation of potentially malignant cells. In contrast, mutations that result in persistent interaction of CAK with TFIIH or mutations that cause a destabilization of the whole TFIIH-CAK complex would be antiproliferative as they would result in reduced levels of activating CDK phosphorylation. The antiproliferative effect of these mutations could explain some of the developmental defects of the *XPD *disorders.

#### The genome instability hypothesis

Changes in chromosome number and structure are commonly observed in tumors, many cancers exhibit aberrant cell architecture, including abnormal centrosomes, multipolar spindles, and breakage-fusion cycles. Chromosomal instability can occur early during tumorigenesis and promotes both tumor progression and heterogeneity [97].

By reducing the ability of cells to repair DNA damage, defects in XP genes result in increased mutation rates, in particular following exposure to UV irradiation. However, similarly increased mutations rates were observed in related syndromes without neoplastic disease. This indicates that the repair defect is necessary, but probably not sufficient to trigger carcinogenesis. It is thus likely that XP mutations also interfere with mechanisms other than DNA repair, thereby causing additional chromosome instability.

Work in *Drosophila *unraveled a role for XPD in maintaining chromosome stability during mitosis in part by regulating the dynamic localization of CAK and thus influencing the kinetics of CDK activity [66]. During the syncytial divisions in *Drosophila *embryos XPD also plays an additional function in the control of the mitotic spindle dynamics, preventing misappropriation of chromosomes by neighboring spindles. This novel role of XPD should also lead to new insights into the molecular basis of the neurodevelopmental defects and the neoplastic disease in XPD disorders.

The function of XPD in ensuring genome stability is conserved in human cells as well, where the TFIIH-independent XPD-containing complex MMXP has recently been implicated in spindle organization and chromosome segregation. Moreover, increased mitotic defects were reported for human cell lines bearing *XPD *mutations leading to the XP and XP-CS phenotype, but not for an *XPD *mutation leading to TTD. While this correlation still needs more extensive testing, these results combined with the *Drosophila *results [66] strongly suggest that chromosome instability is a major factor underlying some of the XP symptoms, in particular the marked cancer predisposition.

#### The aging hypothesis

Malfunctioning of the mechanisms devoted to genetic surveillance is a main cause of human progeroid syndromes, characterized by the precocious appearance of clinical and molecular features of natural aging [98]. Genetically these rare diseases are caused by mutations in genes that are directly involved in DNA repair or in genes encoding lamins A and C or molecules involved in the post-translational processing of lamins [5]. The latter indirectly affect DNA repair by impairing the recruitment of checkpoint and repair factors to the site of damage [99]. Moreover, the progressive loss of integrity of the somatic genome is thought to causally contribute to degenerative aging in normal individuals [93]. As mutagenic lesions may potentially promote cancer, certain types of DNA damage trigger senescence as part of a protective response. Thus aging may be the consequence of compromised tissue functionality due to the accumulation of senescent cells and to the loss of irreplaceable postmitotic cells or, in the case of renewable tissues such as the skin, to the depletion of proliferating stem cell pools [93,100]. Interestingly, mutations in TC-NER specific factors as well as some *XPD *mutations result in increased apoptosis in various tissues. Such increased apoptosis may minimize cancer susceptibility but is also likely to be the main cause of the characteristic progeroid features of these patients.

#### The hormone signaling hypothesis

Additional targets of TFIIH-CAK phosphorylation are members of the nuclear receptor family of transcriptional activators including RARα, RARγ and ERα [31,101]. Mutations in *XPD *that affect the interaction of CAK with TFIIH would result in the failure to properly phosphorylate these nuclear receptors, contributing to the phenotypes of the XPD syndromes, in particular sterility, growth retardation, developmental defects, skeletal and ocular abnormalities.

The retinoic acid receptors (RARs) play critical roles in the development and homeostasis of a variety of tissues. During embryonic development retinoic acid signaling via the RAR receptors is required for patterning and organogenesis, and mutations in RARs or vitamin A deficiency can lead to growth retardation, sterility, ocular defects and skeletal defects [102]. RA signaling is essential for granulocyte differentiation and this may explain some of the immune cell abnormalities in XP. As retinoic acid derivatives have anti-proliferative and pro-differentiation properties, they are employed as preventive treatment and successfully reduce skin carcinogenesis in XP patients [103]. The mechanisms of action of retinoids is unclear, but it may involve non-transcriptional effects that may or may not depend on RARs [104].

Another nuclear receptor phosphorylated by TFIIH-CAK is the estrogen receptor alpha (ERα) [101]. Defects in transcriptional activation via ERα may be causative of some of the observed developmental defects as well as premature aging symptoms. Notably, during normal aging the responsiveness to estrogen decreases. Moreover, estrogen is known to stimulate immune cells and failures of the immune system may be associated with a decreased anti-tumoral response. Also, the sterility of some *XPD *patients could be directly linked to defective estrogen signaling in particular during sperm maturation.

Several postnatal neurodevelopmental processes are regulated by the thyroid hormone through nuclear thyroid hormone receptors. Interestingly, the neurological dysfunctions observed in TTD individuals have strong similarities to thyroid hormone deficiency and deregulated expression of thyroid hormone target genes was observed in the CNS of a murine TTD model [105].

#### The immune system failure hypothesis

Natural killer (NK) cell activity is thought to be important in the immune surveillance against neoplasia. Moreover, since UV-induced tumor cells are generally highly antigenic, they may be lysed by cytotoxic T cells following antigen presentation to helper T cells by Langerhans cells (LC). Unfortunately, besides inducing carcinogenic DNA lesions, UV can also counteract this antitumoral response by exerting immunosuppressive action [106].

Lehman et al. (1989) proposed that the cancer-prone XP phenotype may be caused by reduced antitumoral immune response, possibly as a result of a second recessive mutation [107]. This hypothesis was based on the observation that some XP patients show reduced levels of NK cells in the peripheral blood, in addition to NER defects [108]. Successively, other groups evaluated the phenotype of circulating lymphocytes and the activity of NK cells in XP and TTD patients and in their relatives with variable results. Heterogeneous levels of NK cells were found in most patients and some of their heterozygous relatives and they do not appear to correlate with increased skin cancer lesions [109,110]. Additional defects in the maturation of NK cells may play a role in XP patients, such as reduced secretion of lymphokines by immunoregulatory T cells.

If deregulated NK cell activity is indeed due to an additional recessive mutation in a distinct locus, one would expect to find among the relatives of XP patients individuals with compromised immune response but without the typical XP phenotypes, as well as individuals with NER defects and normal NK cell activity and thus normal antitumoral immune response. This is indeed the case; malignant cutaneous neoplasms develop in (only) approximately 45% of XP patients [8] and immune defects have been reported also in healthy relatives of patients with XP [109,110]. However, it cannot be excluded that impaired NER function in XP patients indirectly affects a pathway required for the differentiation or the activity of immune cells, in particular of NK cells. Notably the DNA damage response triggers the surface expression of NKG2D receptor ligands in precancerous and cancerous lesions. This receptor is expressed in various lymphocytes, including NK cells, and is one of the major receptors required for NK cell mediate lysis of tumor cells *in vitro *[111]. Impaired expression of NKG2D ligands or impaired signaling upon DNA damage may underlie decreased anti-tumor immunity in XP.

### Current models for the study of XPD disorders and function

Various human *XPD/ERCC2 *mutations have been introduced into the corresponding murine gene and the disease phenotypes recapitulate well the human syndromes, although the relative severity of the cancer and neurological defects in specific mouse mutants has been somewhat unpredictable [112,113].

The establishment of a reliable technique to isolate skin fibroblasts from human patients affected by *XPD *syndromes and of several well-established cell-based assays allowed to directly address various molecular aspects of the disease and to compare the relative activity of the mutated alleles and the stability of the mutant proteins. Moreover, it has rendered possible to identify the causative mutation by genetic complementation. Most of these lines are available through the Coriell Institute for Medical Research (Camden, NJ, USA). This will allow in the future to more reliably compare results from different laboratories. Primary fibroblasts from human patients were used mainly to assess the sensitivity to the killing effects of UV irradiation, to study the repair DNA synthesis (or unscheduled DNA synthesis) and the recovery of RNA synthesis after UV irradiation. Recently, cDNA microarray studies were also performed on some of these lines in order to define a possible distinctive transcriptional defect signature that may partially explain the heterogeneity of these syndromes.

A fundamental obstacle to the study of the cellular pathways involving XPD is posed by the concurrence of various processes requiring the presence and/or the function of XPD. In this respect, the *Drosophila *early embryo was used successfully to isolate the cell cycle function of XPD from its requirement during transcription and NER. Using *XPD*-deprived young *Drosophila *embryos it was possible to discover a novel important role for XPD in maintaining genome stability during mitotic divisions. Introducing mutations found in the human diseases into the *Drosophila XPD *gene should therefore reveal which of these mutations affect this particular function and to which extent this may contribute to the disease.

### Implication for the treatment of NER syndromes

One of the most devastating aspect in many NER- and *XPD*-syndromes is the neurological component and understanding, which molecular defects are responsible for neurodegeneration is important for the development of therapeutic strategies. The neuropathology can be very different between syndromes and its causes include neurodevelopmental defects and progressive neurodegeneration (i.e. loss of neuronal cells as seen in XP) and defective myelination (with either reduced levels of myelin or abnormal myelin as observed in CS, COFS and TTD) [114]. For reviews covering the relationship between DNA repair and neurological diseases the reader is referred to specialized reviews [11,50,115]. During the rapid cellular proliferation of neural progenitors genomic stability is of paramount importance as these cells will populate the nervous system where they remain in place throughout the remaining life. Expansion of progenitor cells that incurred mutations or cell loss due to apoptosis (in response to unrepaired DNA damage) could subsequently lead to disease. The possible role of XPD in preserving genomic stability during the early embryonic cell cycles, as seen in *Drosophila*, therefore provides an additional theoretical mechanism for the developmental neuropathology in *XPD *syndromes. In differentiated cells, DNA repair and in particular NER, remain extremely important to maintain the integrity of the nervous system. Damage from endogenous sources is likely to be relevant in causing oxidative and other types of damage that are repaired by NER. Terminally differentiated neuronal cells show reduced GG-NER activity *per se*. Moreover, these cells are exposed to more oxidative damage than other cells because of their high oxygen requirements and in addition they transcribe a large portion of their genome, which makes them more susceptible to subtle defects in transcription on one hand, and to damage dealt with by TC-NER on the other hand. In TTD, CS and COFS the most striking neuropathological feature is abnormal myelination, indicating that oligodendrocytes rather than neurons are affected. In TTD, aberrant myelination is due to the loss of the co-activator function of TFIIH in thyroid hormone-dependent gene expression [105]. In addition, apoptotic cells may induce an inflammatory response resulting in increased oxidative stress and, in turn, in more unrepairable DNA damage. In contrast to neurodevelopmental defects, that are more difficult to approach therapeutically, postnatal neuropathology may in the future be treated. To the extent that oxidative lesions are involved in neurological disease in XPD-syndromes, which still awaits conclusive prove, antioxidants or other drugs that prevent their formation may be useful. Additional degenerative phenotypes reminiscent of physiological aging are likely due to cell death and loss of regenerative potential of various differentiated tissues due to loss of stem cells (subcutaneous fat tissue, hematopoietic stem cells, endocrine cells, various cells of the eye and multiple organs)[7].

One of the main causes of morbidity in XP patients is the development of tumors, in particular skin tumors, because 2 out of 3 XP patients die before reaching adulthood due to metastases. Early diagnosis is important because preventive measures have to be applied immediately after birth. The studies of Fan et al. (2008) and Dubaele et al. (2003) now indicate that impaired XPD helicase activity correlates with phenotypical manifestations characteristic of XP. Assessment of XPD helicase activity or theoretical prediction based on structural data could be standardized as diagnostic strategy. Attempts to attain canceroprotective effects with systemic administration of retinoids (chemoprevention) have been made [103,116]. This therapeutic strategy was rather successful in preventing the development of new skin cancers but unfortunately the benefits ceased when the treatment was discontinued and the long term administration of retinoids is limited by their side-effects. The precise mechanism by which retinoids act as chemopreventive agents in unknown, but many of the wide variety of effects elicited by retinoids are mediated through binding of nuclear receptors. This implies that the beneficial effects of this chemoprevention may be restricted to patients with *XPD *mutations that do not interfere with CAK-dependent activation of these nuclear receptors. A better understanding of the implication of nuclear receptor activation in the patophysiology of XPD disorders is therefore required to select patients for systemic treatment with retinoids. While the primary causative agent of skin neoplasia is certainly UV irradiation, the causes of increased internal cancers are unknown. Internal cancers are most frequently reported in the gastrointestinal trait and lungs, which are more exposed to environmental carcinogens. Thus, these patients may benefit from antioxidants as chemopreventive agents. Finally, while the NER defect is certainly playing a fundamental role in the mechanism of neoplasia in XP patients, the contribution of other XPD-dependent processes still needs to be studied, particularly relevant in this context is the involvement of XPD in cell cycle regulation and maintenance of genome stability.

## Conclusions

The rare human hereditary disorders termed NER syndromes are caused by mutations in genes encoding components of the NER pathway. The most striking symptoms of these syndromes involve two different tissues, namely the nervous system and the skin. Due to defects in the GG-NER, XP patients cannot cope with DNA lesions caused by UV-irradiation and accumulate various abnormalities in exposed tissues such as the eyes and the skin, including precancerous lesions. Such lesions frequently progress to become cancerous lesions because also the apoptotic response to DNA damage is impaired in these patients. In patients of the complementation group D, we propose that additional mechanisms may be implicated in the cancer pathogenesis. XPD impinges on the mechanisms of cell cycle regulation by ensuring that CAK is restrained from phosphorylating its mitotic substrates during the process of DNA repair. Failure to properly arrest the cell cycle in *XPD *mutant cells may result in the expansion of cells that have not terminated the repair of potentially mutagenic mutations, thus contributing to the process of tumorigenesis. In some *XPD *disorders, neurological symptoms add to the neoplastic disease. Some of these neurological symptoms are the consequence of neuronal death resulting from defective repair of oxidative lesions that are frequent in the nervous tissue due to its high oxygen consumption, moreover defective transcription or defective repair of transcribed genes may cause neuronal tissue abnormalities. Finally XP-D patients may be predisposed to neurodegeneration due to the loss of an additional function of XPD in maintaining somatic genome stability. Step by step the molecular and cellular functions of XPD start matching the complexity of the disorders associated with *XPD *and one wonders what other functions still wait to be uncovered.

## Competing interests

The authors declare that they have no competing interests.

## Authors' contributions

EC wrote most of the manuscript and designed all figures; KS and BS contributed to the writing. All authors researched parts of the literature, had intellectual input into the review, and read and approved the final manuscript.
